# Effect of the supergravity on the formation and cycle life of non-aqueous lithium metal batteries

**DOI:** 10.1038/s41467-021-27429-8

**Published:** 2022-01-10

**Authors:** Yuliang Gao, Fahong Qiao, Jingyuan You, Zengying Ren, Nan Li, Kun Zhang, Chao Shen, Ting Jin, Keyu Xie

**Affiliations:** 1 State Key Laboratory of Solidification Processing, Center for Nano Energy Materials, School of Materials Science and Engineering, Northwestern Polytechnical University and Shaanxi Joint Laboratory of Graphene (NPU), Xi’an, 710072 People’s Republic of China; 2 Research & Development Institute of Northwestern Polytechnical University in Shenzhen, Northwestern Polytechnical University, Shenzhen, 518057 People’s Republic of China

**Keywords:** Batteries, Batteries, Engineering, Electrochemistry, Energy storage

## Abstract

Extra-terrestrial explorations require electrochemical energy storage devices able to operate in gravity conditions different from those of planet earth. In this context, lithium (Li)-based batteries have not been fully investigated, especially cell formation and cycling performances under supergravity (i.e., gravity > 9.8 m s^−2^) conditions. To shed some light on these aspects, here, we investigate the behavior of non-aqueous Li metal cells under supergravity conditions. The physicochemical and electrochemical characterizations reveal that, distinctly from earth gravity conditions, smooth and dense Li metal depositions are obtained under supergravity during Li metal deposition on a Cu substrate. Moreover, supergravity allows the formation of an inorganic-rich solid electrolyte interphase (SEI) due to the strong interactions between Li^+^ and salt anions, which promote significant decomposition of the anions on the negative electrode surface. Tests in full Li metal pouch cell configuration (using LiNi_0.8_Co_0.1_Mn_0.1_O_2_-based positive electrode and LiFSI-based electrolyte solution) also demonstrate the favorable effect of the supergravity in terms of deposition morphology and SEI composition and ability to carry out 200 cycles at 2 C (400 mA g^−1^) rate with a capacity retention of 96%.

## Abstract (web summary)

The behavior of lithium metal batteries at various gravity levels is not yet clearly understood. Here, the authors demonstrate the beneficial effect of supergravity in terms of battery performance improvement and favorable solid electrolyte interphase formation.

## Introduction

**Fig. 1:**
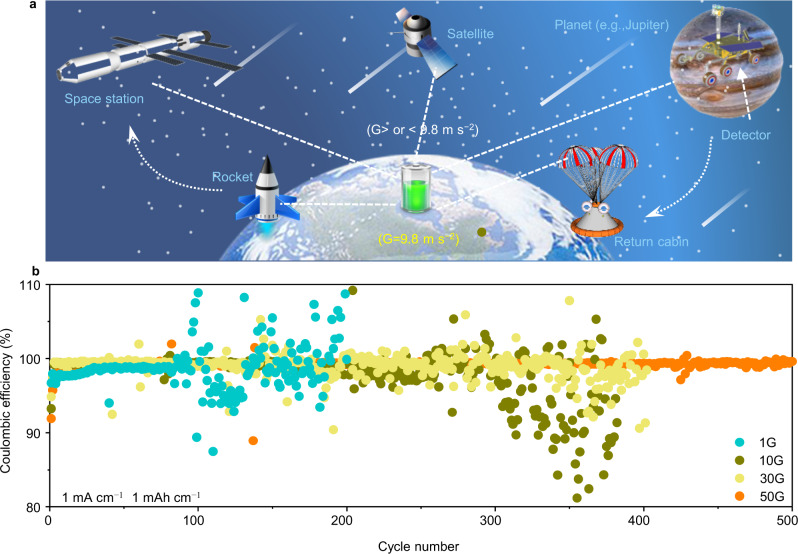
The application background of Li metal batteries and its electrochemical performance under supergravity. **a** Potential applications of Li metal batteries in space exploration. **b** Long-term cycling of Li | |Cu cells with a fixed capacity of 1 mA h cm^−2^ at a current density of 1 mA cm^−2^ under different gravity conditions.

Explaining the formation and evolution of the universe and planets, exploring extraterrestrial life, and establishing permanent human settlements in space are significant reasons why researchers from all over the world are obsessed with outer space^1,2^. Complicated service conditions cause various devices (rockets, satellites, and detectors) to be in short-term or long-term special environments, including microgravity and supergravity (Fig. 1a). In general, supergravity (*G* > 9.8 m s^−2^) is the force of matter greater than that under normal gravity acceleration, while microgravity (*G* < 9.8 m s^−2^) is the opposite^3,4^. As a vital module of space equipment, current aerospace qualified battery technologies must not only adapt to the harsh environment, but also meet the mass and volume requirements of the target mission^5,6^.

Compared with advanced Li-ion batteries with the same energy density, Li metal batteries are more compact due to their lower mass, volume, and capacity^7,8^. Such high specific energy property can not only reduce the detection cost of the transmitter, but also reserve more mass and space for other necessary materials and equipment, and at the same time provide a longer cruising range for specific equipment. Therefore, Li metal batteries have tremendous potential in future space exploration. However, the current research and evaluation are limited to normal gravity^9–15^. A fundamental understanding of how the Li metal batteries behave over a wide range of gravity, like over 1G, is ambiguous.

Supergravity occurs in special planets (e.g., Jupiter, Saturn, and Neptune) and detection carriers that accelerate ascent or decelerate. Jupiter, one of the eight planets in the solar system, is part of NASA’s space exploration program, which began with the launch of the first dedicated Galileo probe in 1989 and again in 2011 with the launch of the Juno probe^16,17^. The China Space Administration is also expected to start a Jupiter exploration program in 2030^18^. In addition, NASA is planning to launch a probe to explore Saturn in 2026 and to start the Neptune exploration program within the next 20 years^19^. Obviously, understanding the working mechanism of materials and equipment under supergravity is of great significance to the smooth development of the planetary exploration program. Supergravity is commonly obtained on the earth by circulating centrifugal force. Under supergravity, centrifugal acceleration approaches to 1000 times gravity acceleration of 9.81 m s^−2^, diphase dynamic factor is substantially raised, and relative speeds of different constitutes in solution are markedly increased^20^. Therefore, supergravity plays an active role in enhancing mass transfer process, momentum transfer, and energy transmission, gaining great attention in material separation, rectification, especially electrochemical deposition^21,22^. Specifically, supergravity was employed in the electropolymerization of the conductive polymers to obtain higher stability and conductivity^23^. Unlike normal gravity, nickel-metal electrodeposition exhibits a distinctive deposition behavior and morphology under supergravity^24^. Furthermore, amorphous tungsten-nickel alloy films exhibited good catalytic activity for hydrogen evolution reaction under supergravity^25^. Essentially, the working mechanism of the Li metal electrode is a classic electroplating and stripping process, meaning that supergravity may impact the fundamental electrochemical behavior of Li metal.

To validate this conjecture, herein, we introduced supergravity during the operation of Li metal batteries and revealed the electrochemical behavior of Li metal in detail. Unlike normal gravity, Li metal electrodes exhibit some new electrode behaviors under supergravity. Specifically, morphological observations indicate that supergravity refines Li particles and presents a dense dendrite-free morphology. Meanwhile, the results reveal that supergravity changes the solvation structure and binding energy of the electrolyte, and enhances the interaction between Li^+^ and anions, which forms a favorable anion-derived inorganic-rich solid electrolyte interphase (SEI). As a result, the Li | |LiNi_0.8_Co_0.1_Mn_0.1_O_2_ (NCM811) pouch cell (2.5 Ah, 325 Wh kg^−1^) shows a stable cycling life of 200 cycles at 2 C (400 mA g^−1^) rate. The performance proves the feasibility of Li metal batteries in supergravity applications and also provides meaningful guidance for research under microgravity.

## Results

### Performance evaluation under supergravity

To explore the influence of supergravity on the electrochemical performance of Li metal batteries in future space exploration, Li | |Cu cells were first employed for evaluation. Supergravity was supplied by rotating centrifugal force, which is a typical practice at present^26,27^, and the coefficients (1G, 1G, 30G, and 50G) and direction (parallel and perpendicular to the electric field) of supergravity are adjusted by rotational speed and the fixed angle of the battery (Supplementary Fig. 1a, b and Supplementary Video). Note that *G* value equal to 1 indicates normal gravity condition, abbreviated as 1G, and the rest are analogous. Fig. 1b compares the Coulombic efficiency (CE) of Li | |Cu cells at a current density of 1 mA cm^−2^ with a capacity of 1 mA h cm^−2^ under different gravity coefficients. For the cells cycled at 1G, the CE at the early stage can rebound to 99.1%, but fluctuates after 84 cycles, which could be attributed to the repeated growth/dissolution of Li metal. Increasing the gravity coefficient to 10G, the CE fluctuates gradually after 250 cycles, while the CE remains stable for the cells cycled at 30G. Further raised to 50G, the cell exhibits a fairly high and stable CE of 99.3% for 500 cycles, showing good cycle stability and Li utilization. The higher and stable CE of the Li metal electrode indicates the uniform deposition of Li under supergravity, and the formation of a stable SEI that can suppress parasitic reactions during the repeated plating/stripping. When applying a much higher current density (3 mA cm^−2^, Supplementary Fig. 2), such an enhanced effect is still noticeable. The rapid CE decay and cell failure can be ascribed to the formation of dead Li (i.e., Li metal regions which are electronically disconnected from the current collector) and unfavorable Li metal depositions (e.g., dendrites).

Li | |Li symmetrical cells were further applied to evaluate the performance of Li metal electrodes under supergravity. At a current density of 1 mA cm^−2^ (Supplementary Fig. 3a), for the cell cycled at 1G, the plating/stripping overpotential was initially maintained at 140 mV and stepped up gradually to 362 mV after 90 h. Subsequently, a sudden drop in overpotential was detected, followed by appreciable voltage fluctuation, which could be explained as the internal soft shorting of the cell owing to the Li dendrites formation^28^. When the gravity coefficient is 10G, the lifespan (210 h) is longer than that of the control group (90 h), but poorer than the cell cycled at 30G (470 h). Further increases to 50G, a lifespan of more than 600 h with a smallest polarization of 30 mV was achieved. At a high current density of 3 mA cm^−2^ (Supplementary Fig. 3b), the cell is cycled for 50 h at 1G before failure, and presents a large polarization voltage during the whole operation process. By contrast, the cycle life of the cells with gravity coefficients of 10G, 30G, and 50G are 1.8, 4.1, and 6.2 times that of 1G, respectively. The performance shows Li plating with a decreased diffusion barrier and realizes uniform deposition. Both Li | |Cu cells and symmetrical Li | |Li cells also show good performance under the perpendicular supergravity direction, further verifying the positive impact of supergravity on the electrochemical performance of Li metal batteries (Supplementary Fig. 4).

### Electrodeposition morphology of Li metal under supergravity

**Fig. 2:**
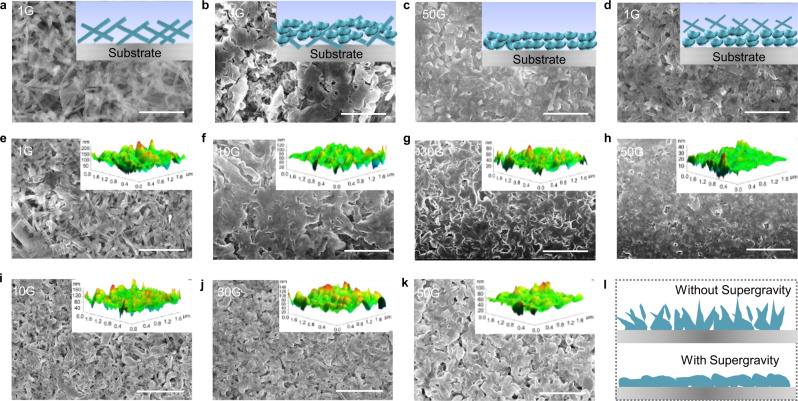
Morphology characterization of Li metal under different gravity conditions. SEM images of deposited Li on Cu after plating 0.5 mA h cm^−2^ at **a** 1G or **c** 50G, and then continues to plate 0.5 mA h cm^−2^ at **b** 50G or **d** 1G. SEM images of deposited Li on Cu after plating 1 mA h cm^−2^ under **e**−**h** parallel and **i**−**k** perpendicular supergravity directions. **l** Schematic illustration of electrodeposition morphology under different gravity conditions. Note that the images inserted in the upper right corner are used to illustrate the corresponding morphological evolution, including **a**−**d** sketches drawn and **e**−**k** 3D topography from AFM. Scale bars are 5 μm in (**a**−**k**).

To reveal the potential mechanism behind the improvement of electrochemical performance, scanning electron microscopy (SEM) was utilized to provide detailed morphology under different supergravity scenarios. Similar to cells with ester-based electrolytes^29,30^, dendritic formations are distributed on the Cu surface after using 1.0 M lithium hexafluorophosphate (LiPF_6_)–ethylene carbonate/diethyl carbonate/ethyl methyl carbonate (EC/DEC/EMC) electrolyte to plate 0.5 mA h cm^−2^ at 1G (Fig. 2a and Supplementary Fig. 5a). On this basis, continue to deposit to 1 mA h cm^−2^ at 50G (Fig. 2b and Supplementary Fig. 5b), and the upper layer of the deposition morphology presents a favorable nodular structure while the lower layer has a clear dendritic morphology, which implies that the supergravity inhibits the subsequent growth of Li dendrites. When 0.5 mA h cm^−2^ is first deposited on the Cu foil at 50G, the tiny nodular morphology appears (Fig. 2c and Supplementary Fig. 5c), indicating the positive effect of supergravity on the inhibition of Li dendrites. Then continues to deposit to 1 mA h cm^−2^ at 1G (Fig. 2d and Supplementary Fig. 5d), the dendrites reappear but better than the deposition morphology under normal gravity only. Unlike the long-lasting effect provided by customized SEI^31,32^, although a favorable SEI forms in supergravity, the rougher electrode accelerates the irregular growth of Li metal at the protrusion (distorted electric field lines), resulting in SEI rupture that cannot regulate subsequent growth. Moreover, the slow mass transfer after the withdrawal of supergravity is not conducive to the uniform deposition of Li metal. To clarify the influence of supergravity on the entire nucleation and growth process of Li dendrites, different deposition capacities (0.2, 1, and 4 mA h cm^−2^) at a current density of 1 mA cm^−2^ were selected. For a small amount of deposition (0.2 mA h cm^−2^, Supplementary Fig. 6), Li metal exhibits a sparse, short fibrous structure at 1G, which is in agreement with the reported result^33^. However, the initial deposition morphology of Li metal turned sharply under supergravity, the unfavorable short fibrous morphology disappeared, and evolved into a more favorable morphology. Specifically, with the increase of gravity coefficient, the initial fibrous structure gradually evolved into a short rod-shaped structure, and oval-shaped structure. As shown in Fig. 2e, due to the tip effect of Li dendrites, at 1G, the deposition capacity continues to 1 mA h cm^−2^, and the surface of the Li metal electrode showed messy Li dendrites with a length of several micrometers. In contrast, the growth of Li dendrites was eliminated under supergravity, and the larger the gravity coefficient, the denser and smoother the deposition morphology (Fig. 2f–h). As shown in inserted 3D topography from atomic force microscopy (AFM), the deposition morphology of Li metal varies from 0 to 200 nm and there are numerous gullies at 1G, indicating the formation of Li dendrites, which provides convenient conditions for the parasitic reaction between Li metal and the electrolyte, and ultimately causes the rapid failure of the cell. As a comparison, under supergravity, the electrode surface becomes smoother and more uniform, and the maximum height is reduced to 120, 80, and 40 nm at 10G, 30G, and 50G respectively. The cross-sectional SEM images further show that supergravity reduces the growth of porous Li to obtain a uniform deposited layer (Supplementary Fig. 7). Further increase to 4 mA h cm^−2^, the Li dendrites still exist at 1G, while the dense and smooth deposition morphology can be maintained under supergravity (Supplementary Fig. 8). Under perpendicular supergravity direction, Li metal also shows favorable deposition morphology (Fig. 2i–k and Supplementary Fig. 9), even at a high deposition capacity (Supplementary Fig. 10), but the morphology is slightly worse to parallel supergravity direction. It can be seen that supergravity can effectively inhibit the dendrites growth and endow the Li metal electrode a uniform and dense morphology (Fig. 2l).

### Intrinsic mechanism of morphology evolution under supergravity

**Fig. 3:**
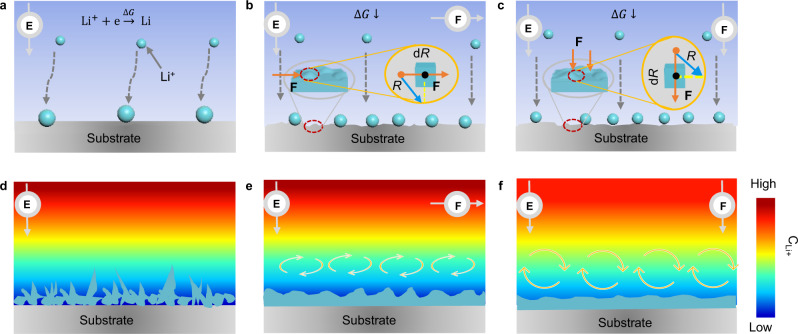
Morphological evolution of the Li metal depositions. Schematic illustrations of the nucleation and growth of Li metal under **a**, **d** 1G gravity, **b**, **e** perpendicular and **c, f** parallel supergravity conditions. Note that “**E**” represents the applied electric field, “**F**” represents the applied supergravity, and the color change represents the difference in ion concentration in the solution.

Regarding the refining behavior of Li particles under supergravity and the difference in deposition morphology under different supergravity conditions, a detailed explanation can be given from the perspective of nucleation and growth. For the nucleation stage, due to the lithiophobic of Cu, the nucleation energy barrier of Li metal is high under normal gravity condition, resulting in a larger nucleus size and a smaller nucleation density, which also provides a prerequisite for the subsequent sparse dendritic morphology (Fig. 3a). When supergravity is introduced in the electrodeposition process, the nucleation energy barrier is reduced, promoting the nucleation of Li metal. To illustrate this phenomenon, we conducted a detailed theoretical analysis from the perspective of electrocrystallization. Under normal gravity, as shown in Eq. (1), the free energy of the system (Δ*G*) is the sum of bulk-free energy and surface free energy. After applying supergravity, the free energy induced by supergravity is newly added, and the Δ*G* is shown in Eq. (2)^34,35^:

1 $$\triangle G=-\frac{4}{3}\pi {r}^{3}\triangle {G}_{V}+4\pi {r}^{2}{\sigma }_{{ls}}$$

2 $$\triangle G=-\frac{4}{3}\pi {r}^{3}\triangle {G}_{V}-\frac{4}{3}\pi {r}^{3}k\varepsilon {{{\bf{F}}}}+4\pi {r}^{2}{\sigma }_{{ls}}$$

Where $r$ is the radius of the crystal nucleus, $\triangle {G}_{V}$ is the free energy change per volume, $k$ is the conversion coefficient, $\varepsilon$ is the bulk shrinkage, ${{{\bf{F}}}}$ is supergravity, ${\sigma }_{{ls}}$ is the surface energy of the Li-electrolyte interface.

To clearly explain the change of free energy under supergravity, a microvolume element (d*R*)^2^ is taken on the surface of the electrode, and its mass center is on the *R* − d*R*/2 plane. Since the value of d*R* is extremely small, it can be dealt with that *R* − d*R*/2 = *R*. The supergravity (${{{\bf{F}}}}$) and radial pressure (${{{\bf{p}}}}$) generated when the micro-volume element rotates are as follows.

3 $${{{\rm{d}}}}{{{\bf{F}}}}=m{\omega }^{2}R=\rho {({{{{\rm{d}}}}R})}^{3}{\omega }^{2}R$$

4 $${{{\rm{d}}}}{{{\bf{p}}}}={{{\rm{d}}}}{{{\bf{F}}}}/{({{{{\rm{d}}}}R})}^{2}$$

Where $m$ represents the unit volume mass, $\rho$ represents the liquid density. Through the integrated calculation of formulas (1)−(4), ${{{\bf{p}}}}$ and $\triangle G$ can be obtained.

5 $${{{\bf{p}}}}=\rho {\omega }^{2}({R}^{2}-{R}_{0}^{2})/2$$

6 $$\triangle G=-\frac{4}{3}\pi {r}^{3}\triangle {G}_{V}-\frac{2}{3}\pi {r}^{3}k\varepsilon {{{\boldsymbol{F}}}}{\omega }^{2}({R}^{2}-{R}_{0}^{2})+4\pi {r}^{2}{\sigma }_{{ls}}$$

Let dΔ*G*/d*r* = 0, the critical nucleation radius ${r}^{* }$can be obtained.

7 $${r}^{* }=2{\sigma }_{{ls}}/\left[\frac{k\varepsilon \rho {\omega }^{2}\left({R}^{2}-{R}_{0}^{2}\right)}{2}+{\triangle G}_{V}\right]$$

Substituting formula (7) into (6), the critical nucleation work $\triangle {G}^{* }$ under supergravity is as follows.

8 $$\triangle {G}^{* }=16\pi {\sigma }_{{ls}}^{3}/\left\{3{\left[\triangle {G}_{V}+\frac{k\varepsilon \rho {\omega }^{2}\left({R}^{2}-{R}_{0}^{2}\right)}{2}\right]}^{2}\right\}$$

To simplify the calculation, several hypotheses are set: (i) Gibbs free energy formula is obtained under the condition of spontaneous nucleation; (ii) bulk shrinkage $\varepsilon$, surface tension ${\sigma }_{{ls}}$ and $\triangle {G}_{V}$ are independent of supergravity; (iii) *R*_0_ = 0. Therefore, $\triangle {G}^{* }$ can be simplified to the following formula.

9 $$\triangle {G}^{* }=16\pi {\sigma }_{{ls}}^{3}/\left[3{\left(\triangle {G}_{V}+\frac{k\varepsilon \rho {\omega }^{2}{R}^{2}}{2}\right)}^{2}\right]$$

From Eqs. (7) and (9), it is known that the greater the rotation speed $\omega$, that is, the larger the gravity coefficient, the smaller the $\triangle {G}^{* }$, which is more beneficial to the crystallization of Li metal. Besides, the reduction of ${r}^{* }$ at a high gravity coefficient means the formation of a small and dense Li nuclei. As observed in the ether-based electrolyte (Supplementary Figs. 11 and 12), the size of the Li nuclei decreases under supergravity and is inversely proportional to the gravity coefficient. Meanwhile, the density of Li nuclei under the parallel supergravity direction is greater than that of perpendicular supergravity direction, and is more pronounced at high gravity coefficients. Such difference may be attributed to the difference in force and active sites on the electrode surface. In the perpendicular direction, the supergravity can only act on the microscopic protrusions of the electrode surface with a partial supergravity component (Fig. 3b), whereas in the parallel direction supergravity can act anywhere on the electrode surface (Fig. 3c), thus providing a wider range of active sites for Li nucleation.

For the growth stage, under normal gravity condition, the slow mass transfer of Li^+^ cannot quickly compensate for the consumption of Li^+^, leading to a steeper concentration gradient near the electrode/electrolyte interfaces, which triggers the growth of Li dendrites (Fig. 3d). Conversely, the convection induced by supergravity accelerates ion mass transfer and relaxes the ion concentration gradient on the electrode surface (Supplementary Fig. 13), ultimately promoting the uniform growth of Li metal without a preferential growth direction. Supergravity-induced convection accelerates the ion mass transfer rate, which follows the following double logarithmic relationship^36^.

10 $${{\log }}{\mu }_{0,G}={{\log }}{\mu }_{0,G=1}+\beta {{\log }}G$$

Where ${\mu }_{0}$ is ion mass transfer rate, $\beta$ is the Tafel slope. Obviously, supergravity is an important factor to adjust mass transport during metal electrodeposition. Although both gravity directions promote ion mass transfer, compare to the horizontal micro-convection induced by perpendicular supergravity direction (Fig. 3e), the longitudinal convection induced by parallel supergravity direction is more beneficial to obtain uniform ion concentration due to the efficient mass transfer behavior (Fig. 3f).

### Effect of the supergravity on the electrolyte structure

**Fig. 4:**
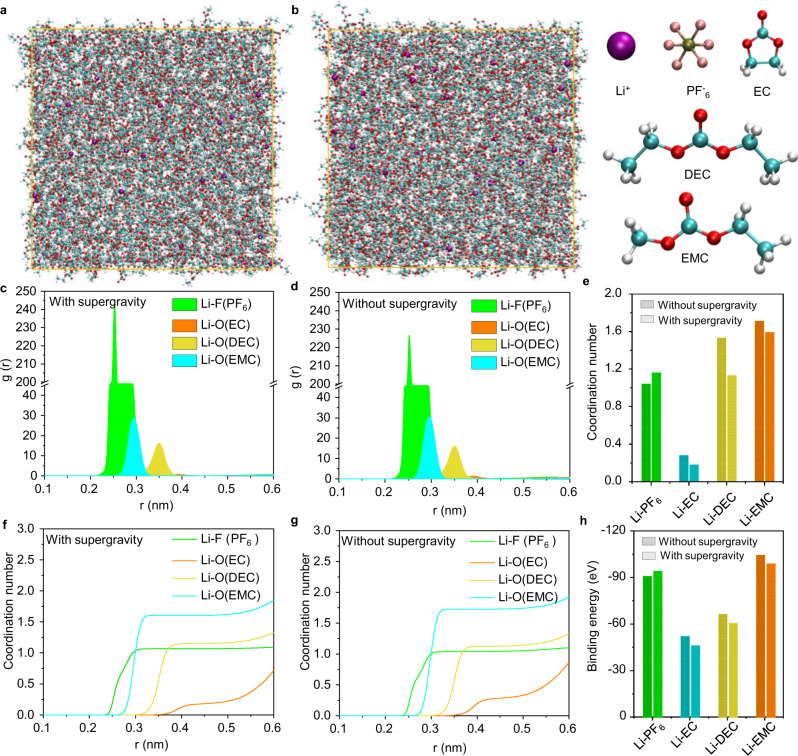
Solvation structures of the liquid electrolyte solution under different gravity conditions. The snapshot of the MD simulated box **a** with and **b** without supergravity. Representative configuration of solvation structures of Li^+^ from MD simulations. RDFs and coordination numbers of Li^+^–PF_6_^−^ and solvent **c**, **f** with and **d**, **g** without supergravity. **e** Comparison of coordination number between Li^+^–PF_6_^−^ and solvent. **h** The binding energy between Li^+^ and various species calculated by the DFT. Color code: Li^+^ purple, P brown, F rose red, C cyan, O red, H white.

In addition to the significant changes in the deposition morphology of Li metal, we also found that supergravity has an effect on the SEI composition. Before revealing the characteristics of SEI, it is necessary to understand the changes in the electrolyte at the molecular level under supergravity. Molecular dynamics (MD) simulations were performed to reveal the solvation structure of the electrolyte under supergravity. The simulation system is constructed with 50 LiPF_6_, 750 EC, 400 DEC, and 500 EMC molecules, and in a cubic box with a dimension of 6.6 nm in length (*x*), width (*y*), and height (*z*). Supergravity is equivalent to giving an acceleration to each atom in the system. As shown in the simulation snapshot (Fig. 4a, b), Li^+^ associate with PF_6_^−^ and solvent molecules, leading to certain solvation structures. The structures can be described by the radial distribution function (RDF), which shows how the density of atom $j$ varies as a function of distance from a reference atom $i$ ($i$ in this case is Li^+^, and $j$ could be either a solvent molecule or PF_6_^−^)^37^.

11 $${g}_{{ir}}(r)=\frac{{N}_{{ij}}(r)/V(r)}{{\rho }_{j}}$$

where ${N}_{{ij}}(r)$ is the ensemble averaged number of atoms $j$ in a spherical shell of volume $V(r)$ at a distance r from atom $i$, and ${\rho }_{j}$ is the bulk density of atom $j$.

The peak in $g(r)$ for Li^+^ appears at around 0.25, 0.29, 0.35, and 0.39 nm for both F atoms in PF_6_^−^ and carbonyl oxygen atoms in EC, DEC, and EMC (Fig. 4c, d), which is consistent with previous reports of LiPF_6_ in ester electrolyte^38^. This indicates that the Li^+^ are solvated by solvents and PF_6_^−^, forming solvation sheaths around Li^+^. Meanwhile, supergravity enhances the correlation between Li^+^ and PF_6_^−^ due to the more pronounced peak intensity. On this basis, the coordination numbers of the PF_6_^−^ and solvent molecules surrounding the solvation shell of the ions are calculated from the following integral.

12 $${n}({R})=4{{{\rm{\pi }}}}{{{{\rm{\rho }}}}}_{{j}}{\int }_{\!\!\!{0}}^{R}{{r}}^{2}{{g}}_{ij}(r){{{{\rm{d}}}}r}$$

Where R is the minimum after the first peak in the RDF ${g}_{{ir}}(r)$. As shown in Fig. 4e–g, when a supergravity is applied, an apparent trend shows that the coordination number of PF_6_^−^ with Li^+^ is enlarges from 1.04 to 1.16 while the coordination number of EC, DEC, and EMC reduce from 0.28, 1.53, and 1.71 to 0.18, 1.13, and 1.59, respectively. The increased anion coordination number strongly implies that PF_6_^−^ anions are more readily to participate in Li^+^ solvation shell. Density functional theory (DFT) calculations further confirmed this conjecture. The binding energy between Li^+^ and PF_6_^−^ is increased from −90.23 to −93.56 eV while weakening the binding energy of other solvent molecules, illustrating a stronger ion association in the electrolyte under supergravity (Fig. 4h).

### SEI formation analysis under supergravity

**Fig. 5:**
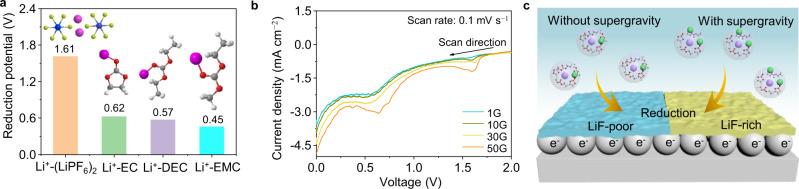
The formation mechanism of SEI under different gravity conditions. **a** Reduction potentials of optimized Li^+^-solvent and (LiPF_6_)_2_ complexes calculated based on quantum chemistry. **b** Typical CV curves of Li | |Cu cells under different gravity coefficients. **c** Schematic illustrations of the SEI formation process. Color code: Li^+^ purple, PF_6_^−^ green, F orange, P blue, C gray, O red, H white.

The altering coordination structures greatly determine the preferential decomposition of electrolyte at the anode-electrolyte interface. Quantum chemistry calculations provide a perspective on electrolyte decomposition. Fig.  5a shows the optimized structures of solvents and salt and the corresponding reduction potentials. It can be seen that the reduction potential of Li^+^–solvent (Li^+^–EC, Li^+^–DEC, Li^+^–EMC) is lower than that of Li^+^–(LiPF_6_)_2_, indicating Li^+^–(LiPF_6_)_2_ will be reduced first during potential decrease. The enhanced association between Li^+^ and PF_6_^−^ under supergravity promotes the formation of more LiF on the surface of Li electrode. As displayed in cyclic voltammetry (CV) curves (Fig. 5b), the sharp PF_6_^−^ decomposition peak (about 1.6 V) is observed in the electrolyte under supergravity, while a relatively weak peak is obtained in the control electrolyte with 1G. Meanwhile, the cathodic peak around 0.6 V for the electrolytes is attributed to the reduction of the carbonate solvent. Based on these, a schematic illustration is presented in Fig. 5c to describe the working scenario of the supergravity strategy. Primarily, the Li surface is negatively charged, which is attributed to the spontaneous escape of Li^+^ from the Li metal surface into the electrolyte. Such negative characteristic leads to the cation enrichment and anion deficiency within the electric double layer due to the electrostatic effect. Therefore, PF_6_^−^ decomposes less under normal conditions, and the SEI compositions are mainly the decomposition products of organic solvents. However, the inherent interaction between Li^+^ and PF_6_^−^ can be enhanced under supergravity, leading to more PF_6_^−^ recruitment into the Li^+^ solvation sheath. These associated solvation clusters are positively charged, allowing more PF_6_^−^ anion present in an electric double layer, which leads to enhanced decomposition kinetics of PF_6_^−^ during SEI construction.

### SEI composition under supergravity

**Fig. 6:**
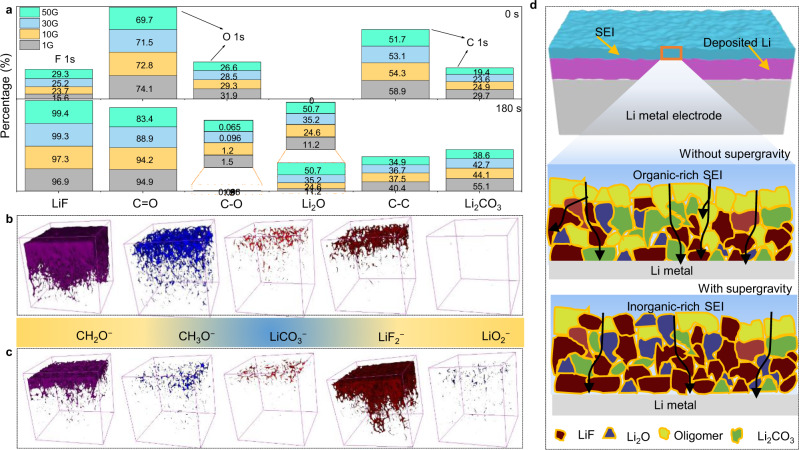
Characterization of the SEI composition under different gravity conditions. **a** Comparison of the percentage of each key component in the SEI to the entire spectrum under different gravity coefficients. 3D views of the element distribution in the TOF-SIMS sputtered volumes of cycled Li electrode with gravity coefficient of **b** 1G and **c** 50G. **d** Schematic illustration of SEI structure on the surface of Li electrode under normal gravity and supergravity.

The distinctive decomposition behaviors of electrolyte under supergravity are bound to affect the composition of SEI. The Li electrodes were collected from the cycled cells under different gravity conditions after 10 plating/stripping for in-depth X-ray photoelectron spectroscopy (XPS) measurements. Fig. 6a compares the percentage of each key component in the SEI to the entire spectrum under different gravity coefficients. The content of LiF increases gradually as the gravity coefficient increases, which is consistent with the intensity of the anion decomposition peak in the CV curves. LiF has been well-known as an SEI component with high interfacial energy and mechanical strength, which can effectively suppress dendrite growth and achieve uniform Li deposition^39,40^. Specifically, the signals of F 1*s* spectra for the four SEI films presented peaks corresponding to LiF (684.9 eV), Li_*x*_PF_*y*_ (687.1 eV), and Li_*x*_PO_*y*_F_*z*_ (688.9 eV), which results from the decomposition of LiPF_6_ salt (Supplementary Fig. 14a, b)^41,42^. After 180 s Ar ion sputtering to remove the surface layer, the LiF peak still exists and the content is higher, indicating its uniform distribution across the depth of SEI. For the O 1*s* spectrum (Supplementary Fig. 14c, d), a peak at 530.2 eV correspond to Li_2_O and two peaks at 533.1 and 531.3 eV correspond to C−O and C=O, respectively^43^. However, Li_2_O has not been detected in the surface of SEI, which only appeared after 180 s etching, and the peak intensity increases as the gravity coefficient increases. This shows that Li_2_O is not present in the surface layer of SEI, and the application of supergravity helps promote the formation of Li_2_O. Note that Li_2_O is an electrically insulating and good ion conductor, which is proven to be beneficial for stabilizing the SEI film^44,45^. Li_2_O usually comes from the native layer of Li metal surface and converts from organic species or Li_2_CO_3_^46^. Besides, the peak intensities of C−O and C=O become weaker under supergravity, which is attributed to the fact that abundant LiF and Li_2_O inhibit the parasitic reaction of Li metal with electrolyte, thereby reducing the decomposition of organic solvents.

For the indicative C 1*s* spectrum (Supplementary Fig. 14e, f), the organic components derived from the decomposition of solvent molecules, which were deconvoluted into four component peaks, namely C−C/C−H (284.7 eV), C−O (286.3 eV), C=O (288.4 eV) and Li_2_CO_3_ (290.1 eV)^47,48^. The top surface of the SEI formed at 1G has a much stronger signal peak, and persists during the subsequent 180 s sputtering, indicating organic compounds are enriched from the surface to the inner part. Clear organic species are also found under supergravity, but this situation is reversed with the increase of the gravity coefficient. After etching for 180 s, the strength of C−C/C−H, C−O, C=O, and Li_2_CO_3_ dropped sharply, which indicates that the organic components and Li_2_CO_3_ were mainly distributed in the outer layer of SEI. Abundant inorganic components effectively inhibit the decomposition of the solvent molecules, which is consistent with the performance of organic components in the O spectrum. Li_2_CO_3_ has a high diffusion barrier and low surface energy for Li^+^, both of which are undesirable in an SEI film to achieve a dendrite-free morphology^49^. Obviously, the reduction of Li_2_CO_3_ content is more helpful for Li metal batteries to obtain excellent electrochemical performance. In addition, the composition of the electrode surface also changed under perpendicular supergravity direction (Supplementary Fig. 15).

The more detailed composition and structure of the SEI were further characterized by time-of-flight secondary ion mass spectroscopy (TOF-SIMS). Based on the depth profiles (Supplementary Fig. 16) and 3D views (Fig. 6b, c) of several fragments, the interfacial structure and components of the Li metal surface can be clearly revealed. Here, CH_2_O^−^, CH_3_O^−^ represent the organic products of electrolyte decomposition, and LiCO_3_^−^, LiF_2_^−^, LiO_2_^−^ represent the inorganic products of electrolyte decomposition, Li_2_CO_3_, LiF, and Li_2_O, respectively. Compared with 1G, the content of CH_2_O^−^, CH_3_O^−^, and LiCO_3_^−^ fragments decreases sharply. The significant contrast mainly stems from the instability of SEI formed at 1G, and the SEI easy to break during charge and discharge, which promotes the parasitic reaction to occur continuously at the rupture of the inner layer of SEI. On the contrary, the content of LiF and Li_2_O increase under supergravity, which greatly stabilizes the structural integrity, prevents parasitic reactions, and reduces the content of organic components. Meanwhile, the organic components are mainly concentrated in the outer layer, serving as the connectors of SEI to withstand the volume change during cycling. Based on the above analysis, schematic illustrations can be obtained as shown in Fig. 6d to depict the distribution features of the components in the SEI. The SEI consists of various components that form a mosaic pattern. For the normal gravity (1G), organic components run through the entire SEI, and a small amount of Li_2_CO_3_, Li_2_O, and LiF are randomly embedded here. For supergravity, the SEI mainly consists of stacked inorganic compounds, where inorganic nanocrystallites are dispersed throughout the amorphous matrix. It mainly displays an abundant distribution of inorganic particles, in which Li_2_O and LiF are more enriched at the metallic Li interface, and an organic layer on the electrolyte side of the SEI.

The unique structure and composition must endow SEI some peculiar properties. There are more organic components in the SEI on the surface of the Li electrode at 1G, resulting in a low Young’s modulus of SEI of only 1.35 GPa, which is consistent with reports in the literature^50^. LiF and Li_2_O are typical inorganic components of SEI, with exceedingly high Young’s modulus, 64.9 and 169 GPa, respectively^51^. Abundant LiF and Li_2_O can significantly enhance the mechanical strength of SEI and prevent the growth of dendrites. As expected, Young’s modulus of SEI shows an escalation tendency when the gravity coefficient is 10G, 30G, and 50G, which are 1.86, 2.98, and 4.21 GPa, respectively (Supplementary Fig. 17). The high mechanical strength can inhibit volume expansion and maintain the structural stability of the SEI during the charge and discharge. The interface ion transport behavior of SEI was further evaluated. The SEI resistance (*R*_SEI_) is significantly decreased under supergravity compared with the normal gravity conditions (Supplementary Fig. 18a and Table 1). The decrease of R_i_ is attributed to the increase in the content of LiF and Li_2_O, which can facilitate space charge accumulation, enhance Li^+^ migration, and reduce electron tunneling, thus leading to a high ionic conductivity and a low electronic conductivity of the SEI. Meanwhile, the increased exchange current density also confirms that SEI under supergravity has excellent interfacial ion transport property (Supplementary Fig. 18b). In short, under normal gravity, the SEI with highly resistive organic species is not conducive to Li^+^ diffusion and easily induces charge accumulation, which ultimately aggravates the formation of dendrites. On the contrary, the SEI formed under supergravity is rich in LiF and Li_2_O, which can provide more ion channels to reach the electrode surface uniformly, thus helping to form a uniform morphology.

### Practical feasibility assessment of full cells under supergravity

To evaluate the service performance of Li metal batteries under supergravity, Li foil was paired with a LiNi_0.6_Co_0.2_Mn_0.2_O_2_-based cathode in a coin cell configuration. Take the supergravity with a gravity coefficient of 50G as an example for evaluation. Initially, the cell delivered a similar capacity at 1G and 50G (Supplementary Fig. 19). However, the capacity of the cell decreased steadily from 160 to 101 mA h g^−1^ after 200 cycles at 1G, while highly improved capacity retention at 145 mA h g^−1^ of the cell for 200 cycles was achieved at 50G. We speculate that the performance difference could be attributed to the stable SEI and uniform Li deposition morphology.

**Fig. 7:**
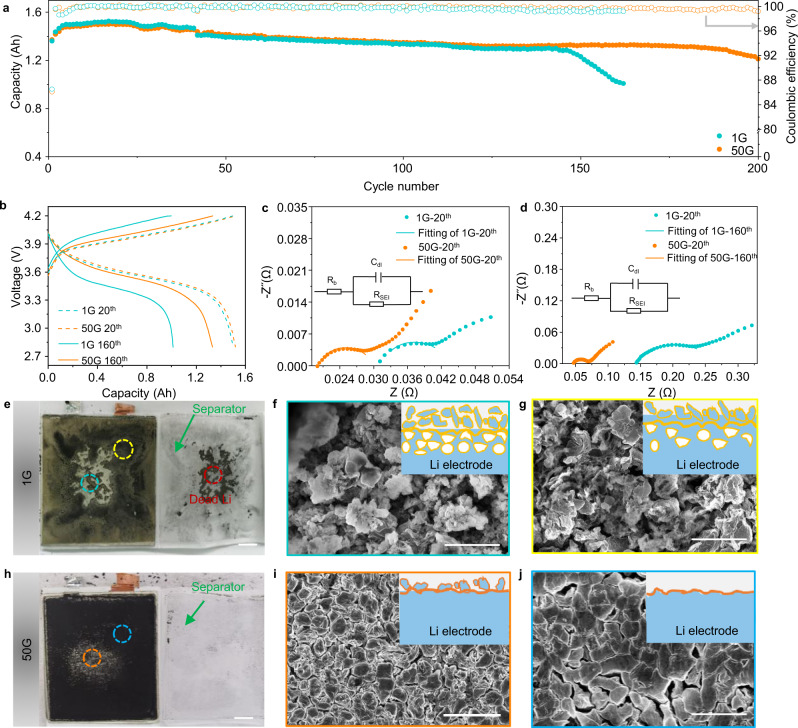
Electrochemical performances of 2.5 Ah high-energy Li | |NCM811 pouch cell under different gravity conditions. **a** Cycling performances of pouch cells at 2 C (400 mA g^−1^) rate. **b** The representative charging/discharging voltage profiles of the pouch cell at 1G and 50G with different cycles. Comparison of impedance spectra of pouch cells after **c** 20 and **d** 160 cycles at 1G and 50G (the inserted equivalent circuit represents the fitted impedance result). **e**, **h** Digital photos and **f**−**j** SEM images of the Li electrode after 160 cycles in pouch cell at 1G and 50G, respectively (the inserted schematic illustrations show the corresponding electrodeposition morphology). Scale bars are 1 cm in (**e**, **h**) and 10 µm in (**f**, **g**, **i**, and **j**).

Limited to actual evaluation defects of coin cells, high-energy Li metal pouch cells were further evaluated under supergravity. In detail, the 2.5 Ah pouch cell with a specific energy of 325 Wh kg^−1^, obtained by coupling a thin Li foil and high-loading LiNi_0.8_Mn_0.1_Co_0.1_O_2_-based electrode (NCM811, 3.84 mA h cm^−2^), was designed and fabricated (Supplementary Fig. 20). The specific energy of the assembled pouch cell is calculated by weighing, and the detailed cell parameters are listed in Supplementary Table 2. As expected, the cycle stability of high-energy Li metal pouch cells under supergravity is better than that in normal gravity. Specifically, the former can cycle steadily more than 100 cycles at 1 C (200 mA g^−1^) rate, while the latter can only keep 80 cycles (Supplementary Fig. 21). Even at 2 C (400 mA g^−1^) high rate (Fig. 7a), the pouch cell exhibited capacity retention of 96% even after 200 cycles at 50G, compared to 84% after 140 cycles at 1G. The performance at high rates may be related to the plating and stripping mechanism of the Li metal electrode and the prevention of parasitic reactions. Such performance is much competitive comparing with other cell-level pouch cells reported from literatures (Supplementary Fig. 22)^52–58^. The charging/discharging profiles of the pouch cell further confirm this distinction in performance (Fig. 7b). The cell showed lower capacity attenuation and polarization at 50G than that at 1G, indicating that the stable SEI and uniform morphology alleviate the parasitic reaction.

Electrochemical impedance spectroscopy (EIS) measurements were carried out to understand the degradation of pouch cells (Fig. 7c, d and Supplementary Table 3). After 20 cycles, the bulk electrolyte (*R*_b_) and SEI resistance (*R*_SEI_) of the pouch cell are only 20 and 8 mΩ at 50G, while the *R*_b_ and *R*_SEI_ of the pouch cells are increased to 31 and 11 mΩ at 1G. The results showed that the pouch cell forms a stable SEI film with high ionic conductivity during the cycle under supergravity, which can not only inhibit the parasitic reaction but also promote the charge transfer, thus alleviating the increase of *R*_b_ and *R*_SEI_. Due to the properties of the SEI under supergravity, the values of *R*_b_ and *R*_SEI_ of pouch cell only increased to 46 and 24 mΩ after 160 cycles at 50G. In contrast, *R*_b_ and *R*_SEI_ of pouch cell increased to 141 and 126 mΩ, about 3 and 5 times that of the former at 1G. Correspondingly, the escalation of impedance is also associated with the rapid failure of the cycle performance of the pouch cell.

Besides, the disassembled pouch cell provides more insights into the decay mechanism. After 20 cycles (Supplementary Fig. 23), there is no noticeable distinction between the optical morphology of Li metal electrodes at 1G and 50G due to the low resolution of the camera, both showing a smooth and dense morphology. Although they also show an approximate morphology under SEM, the surface layer at 1G is thicker than 50G (16 μm vs. 9 μm). After 160 cycles, this diversity is greatly magnified. It was found that the surface of the Li metal electrode became exceedingly rough and incomplete, and some “dead Li” was attached to the separator at 1G (Fig. 7e). On this basis, two different areas were selected for observation on the electrode surface under different gravity conditions. As shown in Fig. 7f, the central area of the electrode presents a porous and loose morphology and contains a large amount of dead Li at 1G, which is also the reason for the adhesion of dead Li on the separator. Compared with the central area, the morphology of other areas has been improved to a certain extent, but the poor morphology has not been fundamentally resolved (Fig. 7g). The worse appearance of the central area may be attributed to the uneven pressure applied by the pouch cell during operation. Such poor SEI cannot stabilize the Li metal/liquid electrolyte interface and regulate the electrodeposition behavior of Li metal, resulting in a torn morphology and a thick cross-section of cycled Li (from 16 to 49 μm, Supplementary Fig. 24a). In contrast, the Li metal electrode can still maintain its original intact morphology at 50G (Fig. 7h). Meanwhile, the Li growth modulated by supergravity is quite stable as shown from the compact and uniform Li plating in the SEM images of Fig. 7i, j. The homogeneous Li layer with a good contract with Li substrate can be more easily removed during the stripping process as reflected by the small amount of dead Li or even no dead Li. Meanwhile, the stable SEI also inhibits parasitic reactions between Li metal and electrolyte under supergravity, resulting in the thickness of the interphase layer only increasing from 9 to 27 μm (Supplementary Fig. 24b). Therefore, introducing supergravity not only facilities stable SEI formation and stabilize the Li metal interfacial reactivity, but also regulates electrodeposition behavior of Li and suppresses dendrite growth, endowing much-improved cycle life in high-energy Li metal pouch cells.

## Discussion

In summary, we creatively considered the gravity factor in the operation of Li metal batteries and revealed the corresponding electrode behavior (deposition morphology, interface properties, and performance) of Li metal. Morphology analysis shows that supergravity can not only inhibit the growth of Li dendrites but also refine Li particles. Moreover, theoretical and experimental results reveal that supergravity enhances the association between Li^+^ and anions, which eventually enables the formation of an anion-derived inorganic-rich SEI. This SEI maintains mechanical stability during cycling and effectively passivates the Li surface, leading to long-term cycling stability. Benefiting from the favorable morphology and SEI, in actual application evaluation, high-energy Li | |NCM811 pouch cell (2.5 Ah, 325 Wh kg^−1^) can steadily run 200 cycles at 2 C (400 mA g^−1^) rate with a retention rate of 96%. This work lights up blind spots on gravity consideration in battery operation, provides a fundamental insight into the effect of supergravity on battery performance, and broadens the operational environments of batteries. Meanwhile, the effect of microgravity on Li-ion batteries is relatively clear, but remains a mystery for Li metal batteries, and further exploration of the mechanisms involved is necessary due to the unique plating and stripping mechanism of Li metal electrodes.

## Methods

### Supergravity experimental equipment

The supergravity is generated by centrifugation derived from a smart agitator. To prevent the power cord from being tangled during the rotation, a conductive slip ring fixed on the ground in the ordinate direction is introduced. The value of gravity coefficient (*G*) can be adjusted by the speed of the agitator and calculated with the following equation:

13 $$G=\frac{{L\omega }^{2}}{g}=\frac{{N}^{2}{\pi }^{2}L}{900g}$$

Where $N$ is the rotation speed, r·min^−1^; $g$ is the gravity acceleration, 9.8 m·s^−2^; $L$ is the distance from the rotary center to the electrode center.

### Physicochemical characterizations

A field emission scanning electron microscope (SEM, Tecnai G2 F30, FEI, Japan) was employed to obtain deposition morphology under different gravity conditions. X-ray photoelectron spectroscopy (XPS, axis Supra, Kratos, Japan) was performed to characterize the chemical composition of the SEI. Atomic force microscope (AFM, Dimension ICON) was used to analyze the roughness and Young’s modulus of the sample surface, and force curves were conducted in the glove box to avoid sample oxidation. AFM tips (BRUKER RTESPA-525) with spring constant of 121 N m^−1^ was used. The value of Young’s modulus can be obtained after fitting by Nova-Px-AFM software. The Time-of-flight secondary-ion mass spectroscopy (ION-TOF GmbH, Germany) was applied to observe the distribution of secondary ions on the electrode surface.

### Electrochemical measurements

CR2016 coin cells were assembled in an Ar-filled glove box with the content of H_2_O and O_2_ below 0.01 ppm. The Li metal chip (purity 99.95%, diameter 1.50 cm, thickness 500 μm) as anode, the Celgard 2500 (polypropylene, porosity 55%, thickness 25 μm, diameter 1.90 cm) as a separator, and 80 μL of electrolyte was added to each cell. The Li | |Cu cells were assembled to evaluate the deposition morphology and Coulombic efficiency of Li metal electrode under different gravity conditions. The electrolyte was 1.0 M LiTFSI dissolved in 1:1 volume ratio of DME/DOL (moisture content is less than 10 ppm). The Li | |Li symmetric cells were assembled with configurations of Li|electrolyte-separator|Li to evaluate plating/stripping performance, and the electrolyte for each cell was 1.0 M LiPF_6_ dissolved in 1:1:1 volume ratio of EC/DEC/EMC (Moisture content is less than 10 ppm). The Li | |NCM622 cells were monitored in galvanization mode within a voltage range of 3.0–4.2 V. Before long-term cycling at the 1 C (180 mA g^−1^), the cells were run at 0.1 C (18 mA g^−1^) for two cycles for better interfacial activation at both anode and cathode. Note that the loading mass of NCM622 is ~13 mg cm^−2^ (thickness 55 μm, diameter 1.20 cm). 1.0 M LiPF_6_ in EC/DEC/EMC (1:1:1, by volume) with 5 wt % fluoroethylene carbonate (FEC) was used as electrolyte (moisture content is less than 10 ppm).

The assembly of pouch cell was performed with a semi-automated cell-manufacturing line in an Ar-filled glove box with the content of H_2_O and O_2_ below 0.01 ppm, which comprises a Z-stacking machine for the cathode, anode, and separator. The pouch cell uses 50 μm Li (purity 99.95%) coated on the Cu foil (thickness 10 μm) as the anode, polyethylene (thickness 9 μm) coated with Al_2_O_3_ (thickness 2 μm) on one side as the separator (porosity 40%), high area capacity LiNi_0.8_Co_0.1_Mn_0.1_O_2_ (NCM811, 3.84 mA h cm^−2^) as the cathode, and contains an in-house prepared electrolyte (4.5 M LiFSI in DME, the moisture content is less than 10 ppm). Note that electrolyte weight to cathode capacity ratio of 2.7 g Ah^−1^. Compared with the conventional 1.0 M LiPF_6_-based electrolyte solutions used in coin cells, the concentrated LiFSI-based electrolyte solution can protect the Li metal electrode and enable the long-term cycling stability of Li metal pouch cells^59,60^. The NCM811 cathode was composed of the active material of 96%, conductive carbon 2%, and binder 2%, and had an area mass loading of about 20 mg cm^−2^ for a single side. To form a stable SEI, the pouch cells were cycled at 0.1 C (20 mA g^−1^) for two cycles and then stored in a 45 °C incubator for 12 h. All pouch cells were tested in the voltage range of 2.8−4.2 V at the rate of 1 C (200 mA g^−1^) or 2 C (400 mA g^−1^). The specific cell parameters for the estimated specific densities are shown in Supplementary Table 2.

All cells (coin cells and pouch cells) were monitored in a LAND multichannel battery cycler (Wuhan LAND Electronics Co., Ltd.) under room temperature (25 °C) without any climatic/environmental chamber. The test of the cell under supergravity or normal gravity is done on the assembled supergravity equipment. In addition, each electrochemical experiment was evaluated 3 times to ensure the validity of the results.

Tafel curves, cyclic voltammetry (CV), and electrochemical impedance spectroscopy (EIS) with different scan rates or voltage ranges were all conducted on a Princeton PARSTAT MC 1000 multi-channel electrochemical workstation. Tafel curves of Li | |Li symmetric cells after polarization at 1 mV s^−1^ from –0.15 to 0.15 V. Typical CV curves of Li | |Cu cells scanned between 0 and 2.0 V at 0.1 mV s^−1^. EIS measurements were performed on the discharge state after 30 min rest time in the frequency range from 100 kHz to 10 mHz with a perturbation amplitude of 5 mV.

### Computational details

The classic molecular dynamic (MD) simulations were carried out in this work using the Gromacs-2021.1 software package. A 10 ns NPT relaxation run at 298 K for the equilibrium MD simulation. The Nose−Hoover thermostat was used to maintain the equilibrium temperature at 298 K and periodic boundary conditions were imposed on all three dimensions. The Particle Mesh-Ewald method was used to compute long-range electrostatics within a relative tolerance of 1 × 10^−6^. A leap-frog algorithm was used with a time step of 1 fs. Supergravity is equivalent to giving an acceleration to each atom in the system. The two systems were simulated for 10 ns, and the data analysis used the 5 ns after the final balance. Quantum chemistry calculations were performed using a Gaussian 09 software package. The reduction potentials were calculated using the same method as the previous works^61^.

## Supplementary information

Supplementary Information

Description of additional Supplementary File

Supplementary Video 1

## Data Availability

The data that support the findings of this study are available within this article and its Supplementary Information. Source data are provided with this paper.

## Source data

Source Data
