# Supplementary material for: Effect of the supergravity on the formation and cycle life of non-aqueous lithium metal batteries
Source: Nat Commun. 2022 Jan 10;13:5. doi: 10.1038/s41467-021-27429-8 (PMC8748458; doi:10.1038/s41467-021-27429-8)
Supplement: Supplementary file 1 — Supplementary Information [file 41467_2021_27429_MOESM1_ESM.pdf]

Supplementary Information

Effect of the supergravity on the formation and cycle life of non-aqueous lithium metal batteries

Yuliang Gao[1], Fahong Qiao[1], Jingyuan You[1], Zengying Ren[1], Nan Li[1], Kun Zhang[1], Chao Shen[1], Ting Jin[1], Keyu Xie[1,2]*

[1]State Key Laboratory of Solidification Processing, Center for Nano Energy Materials, School of Materials Science and Engineering, Northwestern Polytechnical University and Shaanxi Joint Laboratory of Graphene (NPU), Xi'an 710072, P.R. China.

[2]Research & Development Institute of Northwestern Polytechnical University in Shenzhen, Northwestern Polytechnical University, Shenzhen 518057, P.R. China.

Correspondence and requests for materials should be addressed to K. X. (E-mail: kyxie@nwpu.edu.cn).

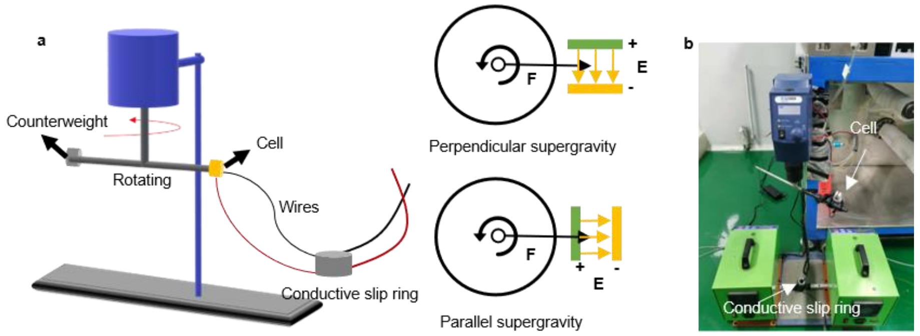

Supplementary Figure 1. **a** Schematic configuration and **b** photographic picture of the experimental equipment. Note that "**E**" represents electric field, and "**F**" represents supergravity.

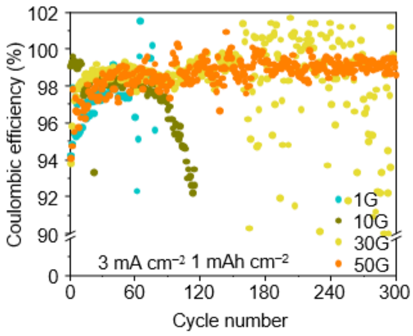

Supplementary Figure 2. Long-term cycle performance of Li||Cu cells under parallel supergravity direction with a cycling capacity of 1 mAh cm^{-2} at the current density of 3 mA cm^{-2}.

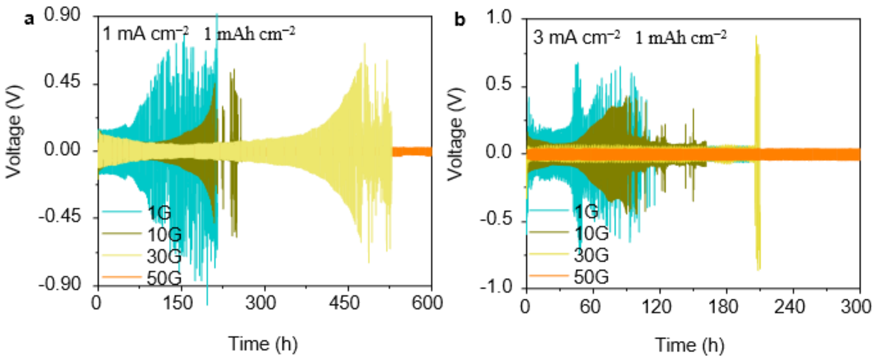

Supplementary Figure 3. Long-term cycle performance of symmetrical Li||Li cells

under parallel supergravity direction with a cycling capacity of 1 mAh cm^{-2} at the current densities of **a** 1 and **b** 3 mA cm^{-2}.

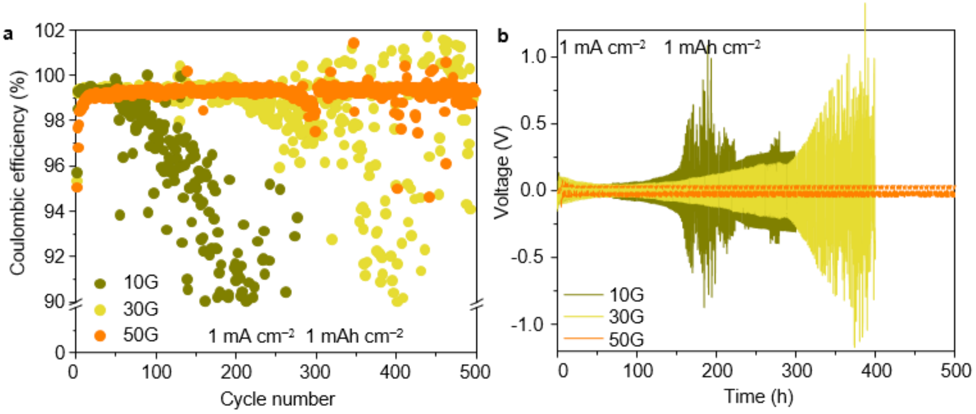

Supplementary Figure 4. Long-term cycle performance of **a** Li||Cu and **b** Li||Li cells under perpendicular supergravity direction.

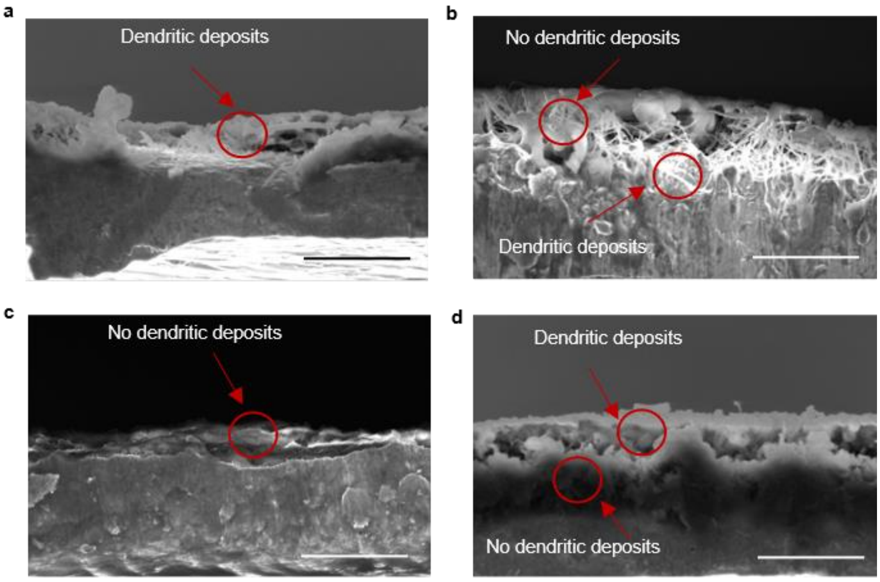

Supplementary Figure 5. The deposition morphology of Li metal under different gravity conditions. Cross-sectional SEM images of deposited Li on Cu after plating 0.5 mAh cm^{-2} at **a** 1G, and then continues to plate 0.5 mAh cm^{-2} at **b** 50G. Similarly,

SEM images of deposited Li on Cu after plating 0.5 mAh cm^{-2} at **c** 50G, and then continues to plate 0.5 mAh cm^{-2} at **d** 1G. Note that the current density of electrodeposition is 1 mA cm^{-2}. Scale bars are 10 μm.

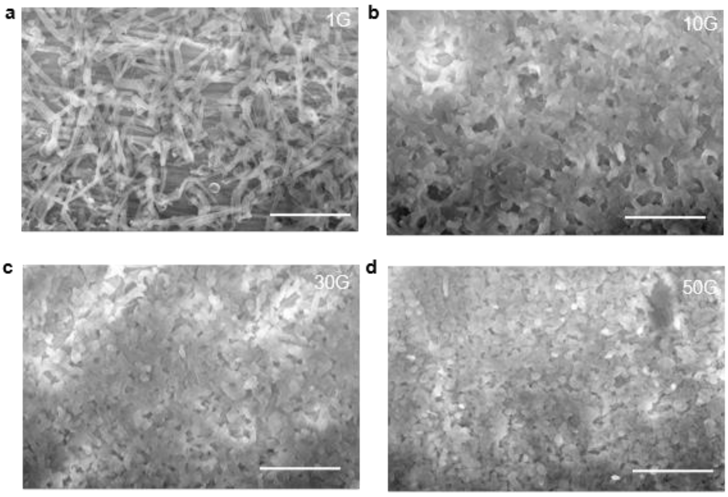

Supplementary Figure 6. Deposition morphology of Li metal in ester-based electrolyte under parallel supergravity direction. Top-view SEM image of deposited Li on Cu after plating 0.2 mAh cm^{-2} with a current density of 1 mA cm^{-2} at **a** 1G, **b** 10G, **c** 30G, and **d** 50G, respectively. Scale bars are 5 μm.

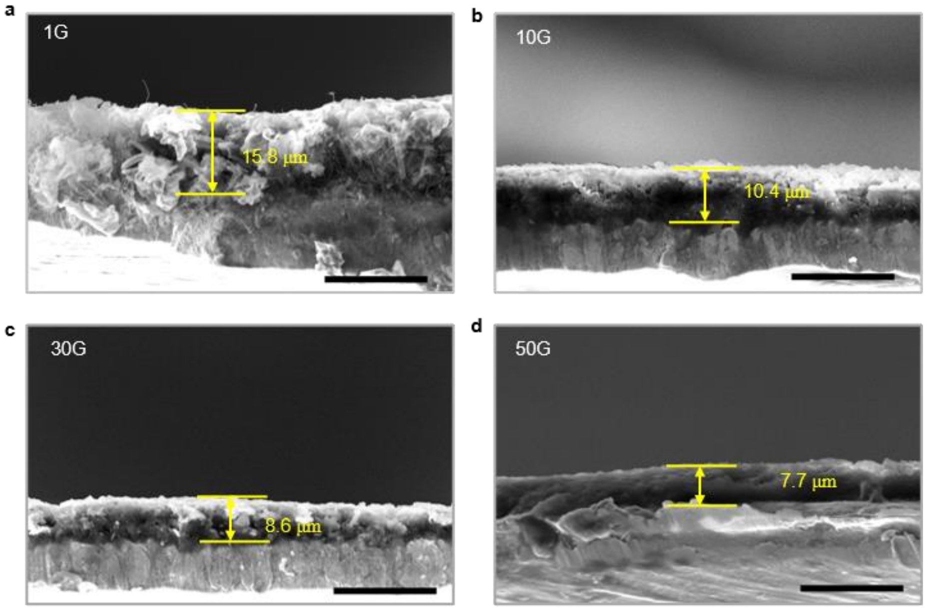

Supplementary Figure 7 Deposition morphology of Li metal in ester-based electrolyte under parallel supergravity direction. Cross-sectional SEM image of deposited Li on Cu after plating 1 mAh cm^{-2} with a current density of 1 mA cm^{-2} at **a** 1G, **b** 10G, **c** 30G and **d** 50G. Scale bars are 20 μm.

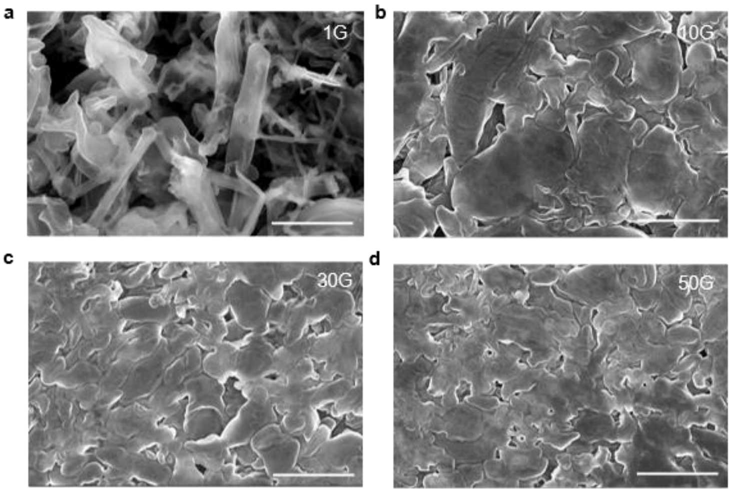

Supplementary Figure 8. Deposition morphology of Li metal in ester-based electrolyte under parallel supergravity direction. Top-view SEM image of deposited Li on Cu after plating 4 mAh cm^{-2} with a current density of 1 mA cm^{-2} at **a** 1G, **b** 10G, **c** 30G, and **d** 50G, respectively. Scale bars are 5 μm.

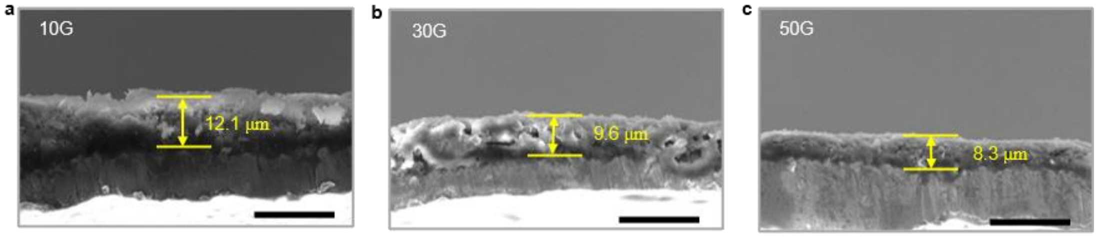

Supplementary Figure 9. Deposition morphology of Li metal in ester-based electrolyte under perpendicular supergravity direction. Cross-sectional SEM image of deposited Li on Cu after plating 1 mAh cm^{-2} with a current density of 1 mA cm^{-2} at **a** 10G, **b** 30G and **c** 50G. Scale bars are 20 μm.

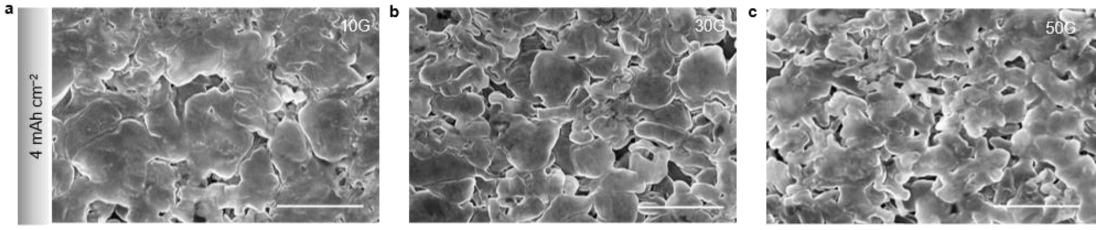

Supplementary Figure 10. Deposition morphology of Li metal in ester-based electrolyte under perpendicular supergravity direction. Top-view SEM image of deposited Li on Cu after plating 4 mAh cm^{-2} with a current density of 1 mA cm^{-2} at **a** 10G, **b** 30G, and **c** 50G, respectively. Scale bars are 5 μm.

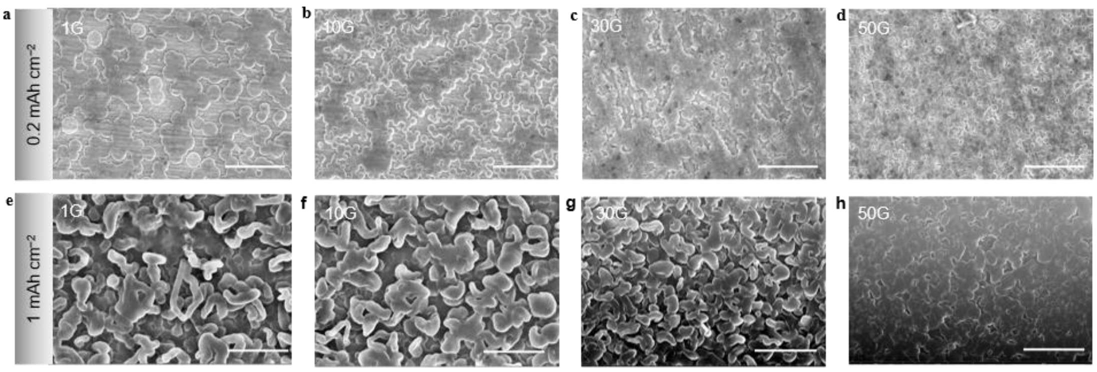

Supplementary Figure 11. Deposition morphology of Li metal in ether-based electrolyte under parallel supergravity direction. SEM images of Li metal deposits at a current density of 2 mA cm^{-2} with deposition capacities of 0.2 and 1 mAh cm^{-2} at **a, e** 1G, **b, f** 10G, **c, g** 30G, and **d, h** 50G, respectively. Scale bars are 20 μm.

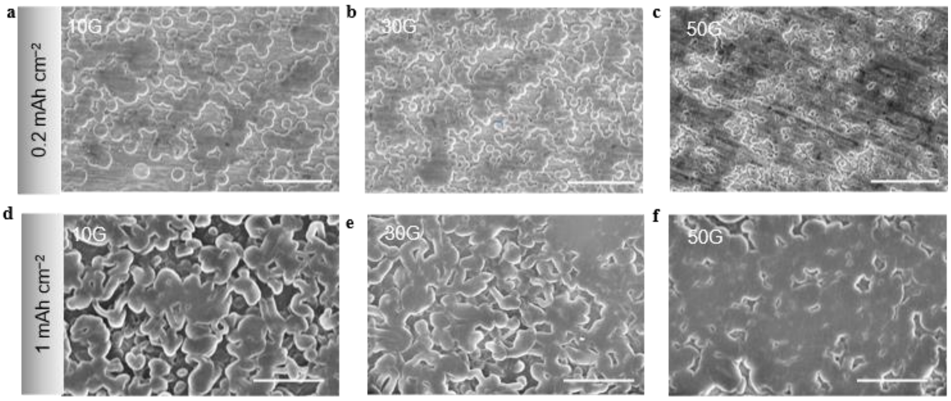

Supplementary Figure 12. Deposition morphology of Li metal in ether-based electrolyte under perpendicular supergravity direction. SEM images of Li metal deposits at a current density of 2 mA cm^{-2} with deposition capacities of 0.2 and 1 mAh cm^{-2} at **a, d** 10G, **b, e** 30G, and **c, f** 50G, respectively. Scale bars are 20 μm.

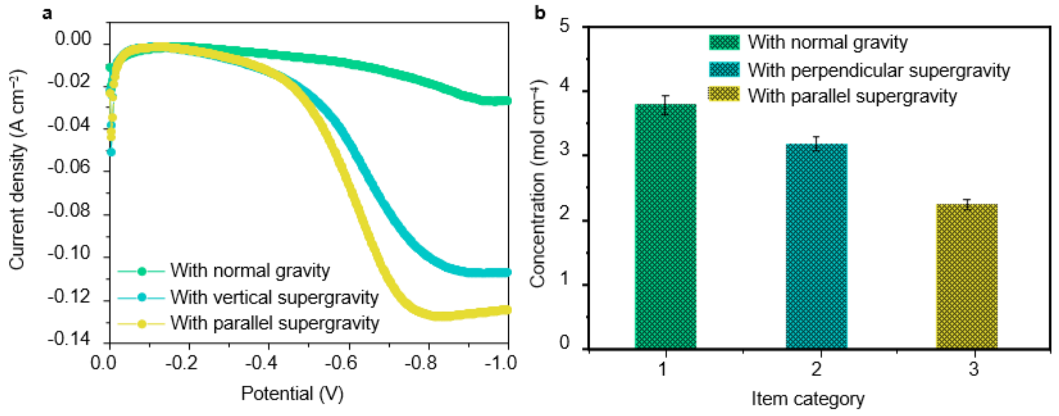

Supplementary Figure 13. **a** The polarization curves of the cells. **b** The ion concentration gradient on the electrode surface under different supergravity conditions. Error bars represent standard deviation, n = 3 independent replicates.

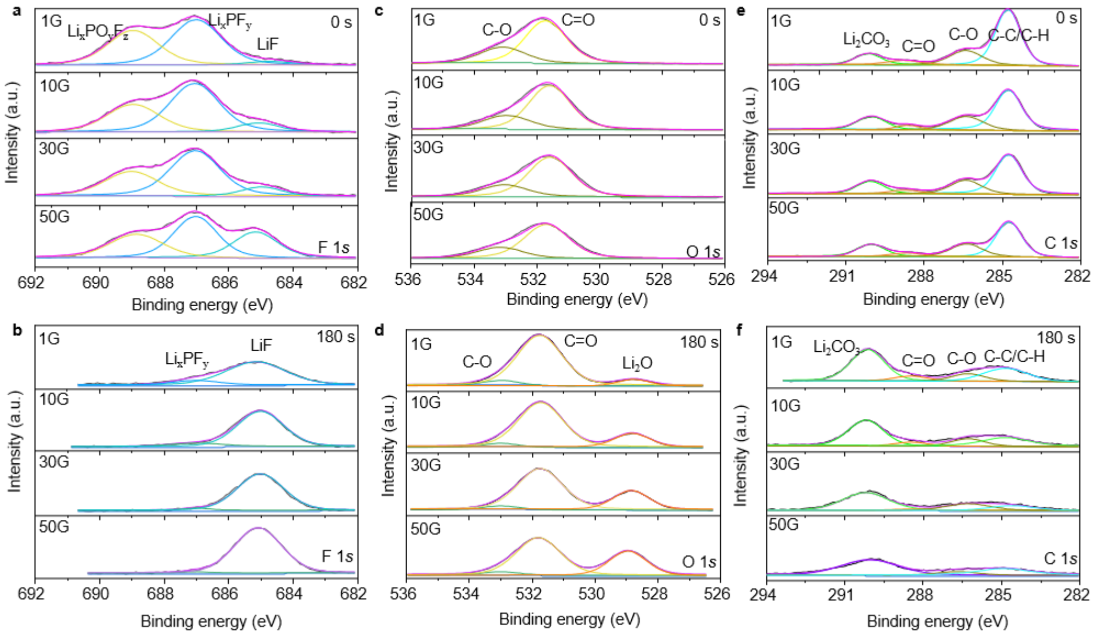

Supplementary Figure 14. XPS spectra of **a, b** F 1*s*, **c, d** O 1*s*, **e, f** C 1*s* for cycled Li metal electrode after etching 0 s and 180 s under parallel supergravity direction.

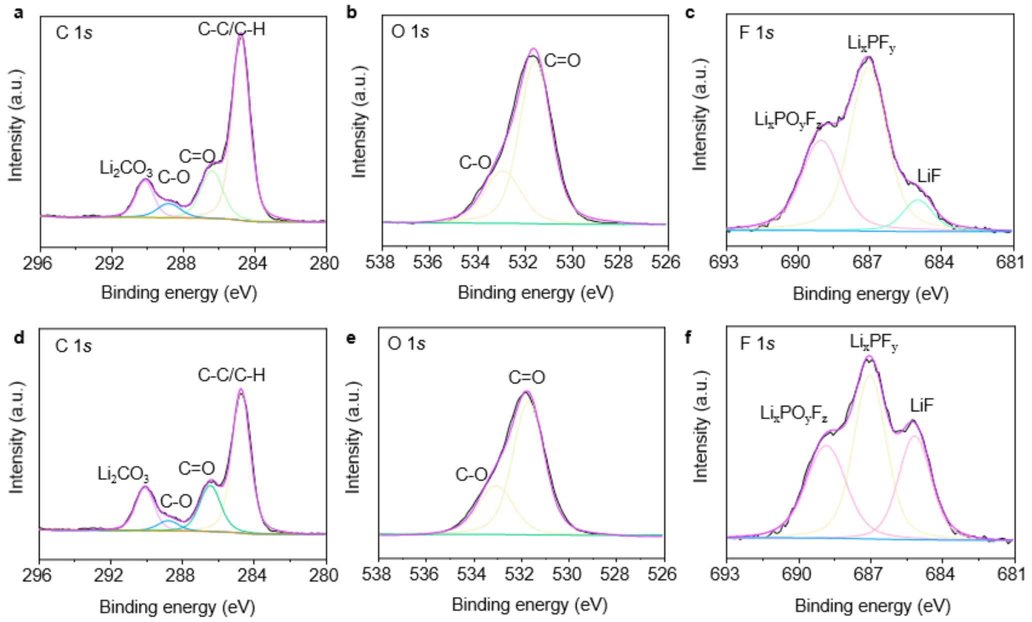

Supplementary Figure 15. XPS spectra of C 1*s*, O 1*s*, and C 1*s* for cycled Li metal electrode under **a–c** normal and **d–f** perpendicular gravity conditions. For the convenience of comparison, the XPS spectra under normal gravity conditions (1G) are from Supplementary Figure 14. Note that the coefficient of perpendicular supergravity here is 50.

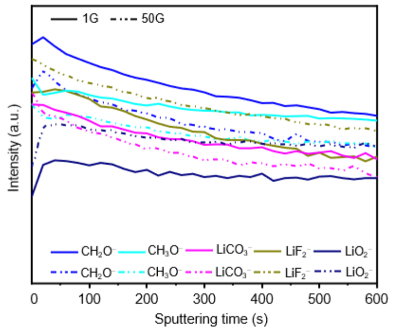

Supplementary Figure 16. Normalized TOF-SIMS depth profiles of a series of secondary ion fragments of interest collected at the surface of the cycled Li metal anode at 1G and 50G.

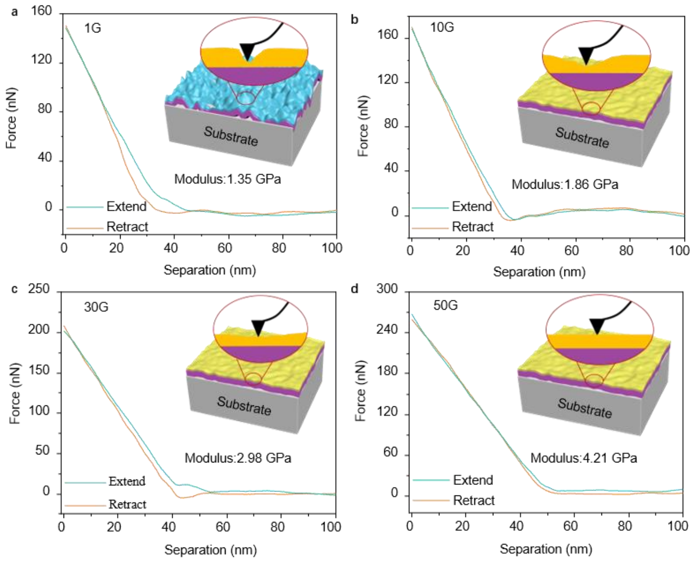

Supplementary Figure 17. The mechanical properties of the SEI. Typical force-displacement curve of Li surface at **a** 1G, **b** 10G, **c** 30G, and **d** 50G. Insert: schematic illustration of corresponding properties of the SEI.

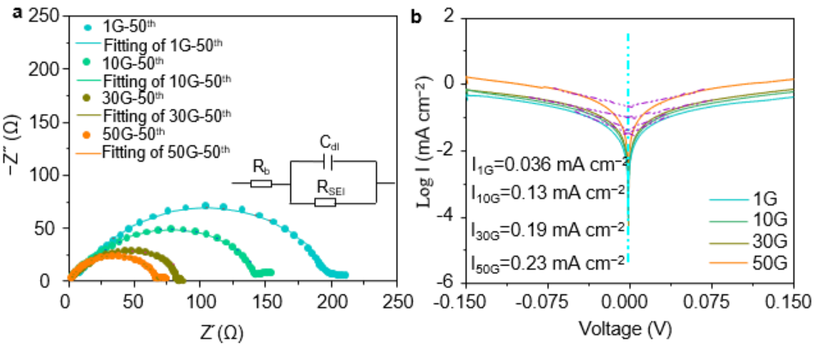

Supplementary Figure 18. Evaluation of ion transport behavior. **a** EIS of Li||Li symmetric cells after 50 cycles under different gravity coefficient (the inserted equivalent circuit represents the fitted impedance result). **b** Tafel curves of symmetric cells after polarization at 1 mV s^{-1} from –0.15 to 0.15 V. The values presented in the EIS are the result of the synergistic effect of the various components of the SEI, including LiF, Li_2O, Li_2CO_3, C=O, C−C, C−O, and other decomposition products

(PO_3^-) of the Li salt. The content of the various substances and the ionic conductivity are different, which means that the contribution to the impedance value is also different and leads to a more complex impedance variation. Based on EIS, the R_{SEI} values of the battery at 30G and 50G are 89.61 and 65.75 Ω, respectively. Although the difference between this value is less than 10G and 30G, it can also reflect the ion transmission characteristics of the interface under these two gravity coefficients.

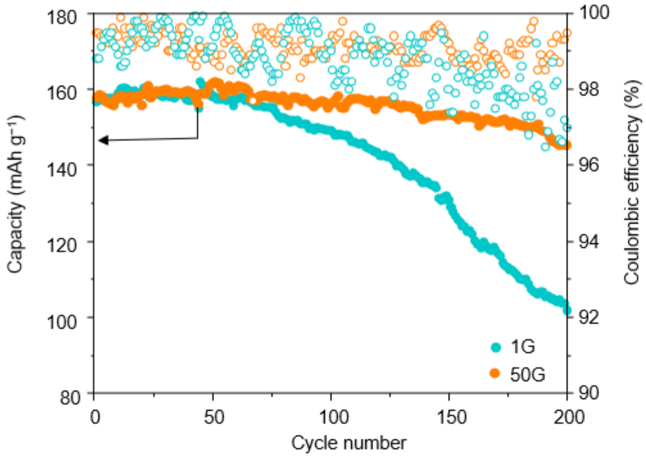

Supplementary Figure 19. Cycle performance of Li||NCM622 coin cell at 1 C (180 mA g^{-1}) rate.

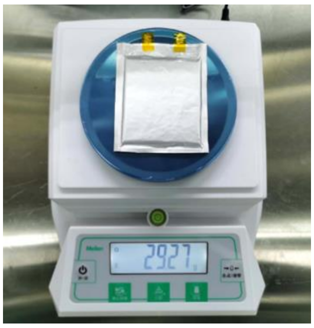

Supplementary Figure 20. Digital photo of the Li||NCM811 pouch cell.

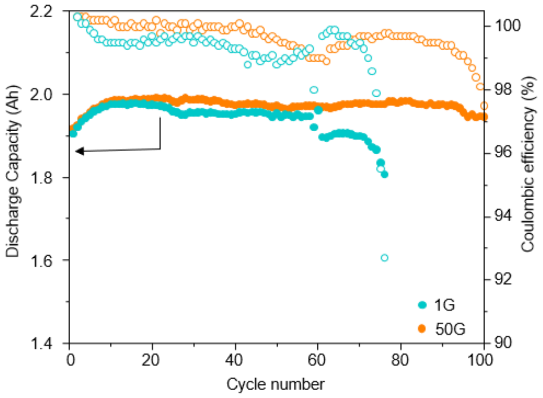

Supplementary Figure 21. Cycle performance of Li||NCM811 pouch cell at 1 C (200 mA g^{-1}) rate.

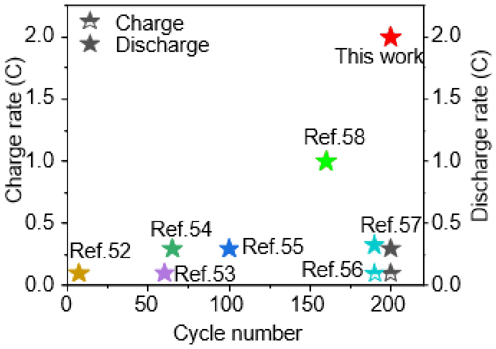

Supplementary Figure 22. Comparison of the cycling performance of pouch cell in this work and previously reported in the literatures.

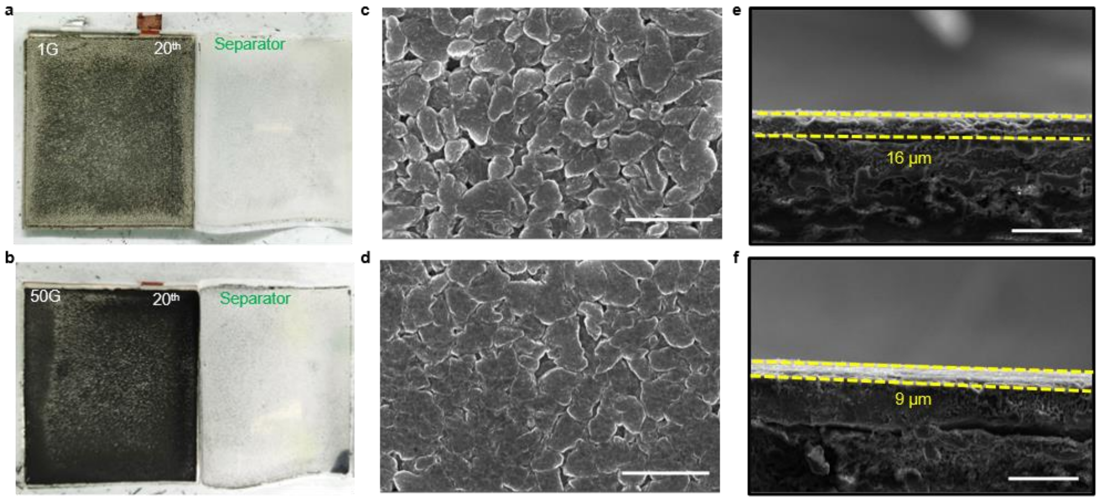

Supplementary Figure 23. Disassembly analysis of pouch cell. **a, b** Digital photos

and **c-f** SEM images of the Li anode after 20 cycles in pouch cell at 1G and 50G, respectively. Scale bars are 1 cm in **a, b**, 10 μm in **c, d** and 50 μm in **e, f**.

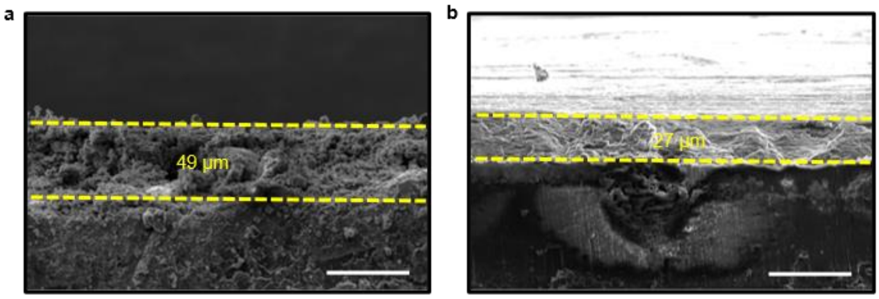

Supplementary Figure 24. SEM images of the Li anode after 160 cycles in pouch cell at **a** 1G and **b** 50G, respectively. Scale bars are 50 μm.

Supplementary table 1. Fitted EIS results of Li||Li symmetric cells after 50 cycles under different gravity coefficient.

Sample	R_{SEI} (Ω)	Error (%)
1G	197.60	0.77
10G	140.60	1.22
30G	89.61	1.45
50G	65.75	0.92

Supplementary table 1. Technological parameters of a Li||NCM811 pouch cell with a specific energy of 325 Wh kg^{-1}.

Cell Component	Cell Parameters	Value
Cathode	Material	NCM811
	Reversible capacity (mAh g^{-1})	200
	Active material loading (%)	96
	Areal weight (each side, mg cm^{-2})	20
	Areal capacity (each side, mAh cm^{-2})	3.84
	Electrode length (mm)	68
	Electrode width (mm)	60
	Number of positive electrode sheets	8
	Aluminum foil thickness (μm)	15
Anode	Material	Li
	Specific capacity (mAh g^{-1})	3860
	Electrode thickness (each side, mm)	50
	Areal capacity (each side, mAh cm^{-2})	10
	Electrode length (mm)	70
	Electrode width (mm)	62
	Number of negative electrode sheets	9
	Copper foil thickness (μm)	10
	N/P ratio	2.60
Separator	Weight (g)	1.21
Packing foil	Weight (g)	1.37
Electrolyte	E/C ratio (g Ah^{-1})	2.70
Tab	Weight (g)	0.21
Pouch cell	Capacity (mAh)	2510
	Weight (g)	29.27
	Specific energy (Wh kg^{-1})	325

Supplementary table 3. Fitted EIS results of pouch cell after 20 and 160 cycles at 1G and 50G.

Sample	R_b (Ω)	Error (%)	R_{SEI} (Ω)	Error (%)
1G-20th	0.031	1.81	0.011	2.56
50G-20th	0.020	1.48	0.008	2.68
1G-160th	0.141	0.70	0.126	3.28
50G-160th	0.046	2.78	0.024	7.26
