# Supplementary material for: Effect of the supergravity on the formation and cycle life of non-aqueous lithium metal batteries
Source: Nat Commun. 2022 Jan 10;13:5. doi: 10.1038/s41467-021-27429-8 (PMC8748458; doi:10.1038/s41467-021-27429-8)
Supplement: Supplementary file 2 — Description of additional Supplementary File [file 41467_2021_27429_MOESM2_ESM.pdf]

Description of additional supplementary data file

File name: Supplementary Video 1
Description: The scene of the battery working on a supergravity device.
